# An overview of approaches for assessing the environmental sustainability of diets – a scoping review for Nordic Nutrition Recommendations 2023

**DOI:** 10.29219/fnr.v68.10453

**Published:** 2024-12-27

**Authors:** Tim G. Benton, Helen Harwatt, Anne Høyer-Lund, Helle Margrete Meltzer, Ellen Trolle, Rune Blomhoff

**Affiliations:** 1 Royal Institute of International Affairs, Chatham House, London, UK; 2 The Norwegian Directorate of Health, Oslo, Norway; 3 Division of Climate and Environmental Health, Norwegian Institute of Public Health, Oslo, Norway; 4 National Food Institute, Technical University of Denmark (DTU), Kgs. Lyngby, Denmark; 5 Department of Nutrition, University of Oslo, Oslo, Norway; 6 Division of Cancer Medicine, Oslo University Hospital, Oslo, Norway

**Keywords:** sustainable food systems, dietary guidelines, environmental impacts, food systems

## Abstract

Assessing the environmental impacts of food, food systems and diets is highly complex due to the multitude of processes involved, the uncertainty in assessment models, the variability in production systems and the large range of products available. No single assessment method alone can provide a complete evidence base. The increasing number of Life Cycle Assessment and food system analyses, and more recently the integration of planetary boundaries offer insights from which we can draw some robust high-level conclusions, whilst recognising there is a need for more detailed analysis to capture the inherent nuances of more location and context-specific situations.

Despite the complexity of assessing the environmental sustainability of food, diets and food systems, there are a number of key considerations that could be used to guide this process, and in doing so, they help to increase utility of the outcomes and limit unintended adverse consequences. We identified five key considerations that can be applied (consider the thresholds, consider the system, consider the variables, consider the context and consider the spillover) to ensure assessments are comprehensive.

## Popular scientific summary

- We identified five key considerations that can be applied when assessing the environmental impacts of food consumption.
- The key considerations are 1) consider the thresholds, 2) consider the system, 3) consider the variables, 4) consider the context and 5) consider the spillover.
- These key considerations will be incorporated into the process of developing guidelines for healthy and environmentally friendly diets in the eight Nordic and Baltic countries.

Through historical development in agriculture and fisheries, the global food system currently produces sufficient food for 8 billion people (1). Partly due to the global food system, global health, total life expectancy and healthy life expectancy have steadily improved over many decades (2, 3). The food system has, however, fundamental flaws that must be addressed to feed current and future global populations in a healthy and sustainable way. A principal focus on increasing productivity of a small suite of agricultural commodities has contributed to the growing problem of malnutrition and over-nutrition through the ‘cheaper food paradigm’ (4).^I^ Global food production does not meet dietary guidelines and is too heavily focused on a small variety of crops and animal products, with too few fruits, vegetables, pulses and nuts leading to too little consumption of plants in diets, too much sugar or other discretionary foods and too much grain used for farmed animal feed (5, 6). Coupled with production insufficient for dietary health, globally, the distribution of foods remains unequal. As a result, malnourishment in all its forms – stunting, hunger, hidden hunger, overweight and obesity – and the diseases associated with it is a global problem, affecting all countries and regions (7, 8). Malnourishment from poor diets results in substantial personal and societal level costs, estimated to be $US3.5tn per year (9). However, this is likely to be an underestimate for several reasons: (1) a lack of data on the public health status of malnourishment, (2) lack of robust accounting and attribution of disease and (3) unequal healthcare spending between countries. In addition to malnutrition, food production has other adverse health impacts including those arising from air pollution. For example, one US analysis found that for every dollar of revenue, around 55 cents of health costs through air pollution was levied (10). In total, the economic impact of ill-health arising from the global food system may exceed 10% of global gross domestic product (GDP) (11).

In addition to not delivering global – or local – public health, the food system has substantial impacts across a range of environmental aspects – both in terms of resource use and adverse outcomes. Globally, around a third of greenhouse gas (GHG) emissions arises from the food system (12), which is also the main user of land, source of nitrogen and phosphorus pollution, and driver of biodiversity loss (13).

The food system contributes substantially to soil degradation, water use and air pollution (13). Adverse impacts of the food system are expected to increase – for example, food-related GHGs will roughly double by 2050 under business-as-usual projections of food production and consumption (14), and more than 17,000 species will be threatened with extinction (15). This increase will further the already burgeoning impact of the food system on earth’s biogeophysical and biogeochemical constraints or ‘boundaries’ related to climate change, land use change, biodiversity loss, and nitrogen and phosphorus pollution – beyond which ecosystem destabilisation is expected to accelerate (16). Without transformative action in the food sector, it will be impossible to meet vital planetary health goals related to climate change and biodiversity (15, 17). This transformative action in the food sector must also consider the distribution of resources, benefits and burdens between countries and regions.

There is no single approach neither for assessing environmental sustainability in the food system nor, indeed, for robustly or comprehensively assessing the sustainability of diets, supply chains or agriculture itself. This relates to both the number or types of environmental metrics to include, the relationship between relative (e.g. intensity of impact per item of food) and absolute (i.e. the aggregated impact of total supply) measures, how to include them in a meaningful way in respect to national and international impacts and contexts and how to consider health and environmental aspects together. Furthermore, there is no single approach to measuring status and progress against sustainability indicators or to assess an overall level of ‘sustainability’ across a range of indicators or sustainability dimensions. The aim of this paper is to provide an overview of the different approaches to assessing the environmental sustainability of diets and identify key considerations to assist comprehensive assessments. We outline the food system within the earth system, and the underlying complexities that subsequently shape environmental assessments. We take a global approach throughout.

The Nordic Council of Ministers (NCMs) has commissioned an update of the Nordic Nutrition recommendations (NNRs). The NNR Committee will develop comprehensive scientific advice to the national health and food authorities who are responsible for formulating the national food-based dietary guidelines (FBDGs) in Denmark, Estonia, Finland, Iceland, Latvia, Lithuania, Norway and Sweden.

The new edition (NNR2023) has been tasked to integrate sustainability aspects into the FBDGs. Sustainability is a complex concept that includes environmental as well as economic and social dimensions. Whilst we acknowledge that all dimensions need careful scrutiny, the remit of this paper is to focus on the environmental dimension of sustainability. This paper focuses on global considerations and, hence, does not consider the local context in Nordic and Baltic countries. This paper will be followed by papers that further explore the environmental sustainability, as well as socioeconomic dimensions, of food production and consumption within the Nordic and Baltic countries (Box 1).

Box 1 The Nordic Nutrition Recommendations.

- This paper is one of many scoping reviews commissioned as part of the Nordic Nutrition Recommendations 2023 (NNR2023) project (18).
- The NNR is commissioned by the Nordic Council of Ministers (NCMs) and the food and health authorities in the Nordic and Baltic countries.
- NNR, which is updated every 8–10 years, is the main scientific framework for nutrition policies, health care, food production, agriculture and fisheries policies, surveillance and research in Denmark, Estonia, Finland, Iceland, Latvia, Lithuania, Norway and Sweden.
- The NNR Committee is appointed by national health authorities in the Nordic and Baltic countries and is responsible for organising the project and publishing the final report with updated dietary reference values (DRVs) and FBDGs.
- More than 200 scientists assisted the NNR committee in the development of the 6th edition of the NNR published in 2023.
- Health effects of nutrients and foods have been the basis for previous recommendations. Based on a request from the NCM, the upcoming edition of NNR will also integrate the environmental aspect of sustainability.
- To assist integration of environmental sustainability, numerous experts from the region were recruited to ensure integration of the national context. In addition, researchers from Chatham House, an independent internationally renowned policy and research institute, were recruited as external experts to secure an unbiased global perspective.
- Whilst the NNR Committee seeks advice from numerous cross-disciplinary scientists, it is the sole responsibility of the NNR Committee to formulate FBDGs and DRVs.
- NNR provides scientific advice to national authorities in the Nordic and Baltic countries. Following the publication of the new edition of NNR, it is the responsibility of the national authorities to formulate national recommendations and guidelines.
- Self-sufficiency and food security will be touched upon in NNR. It is, however, outside the scope of NNR to integrate such aspects. Where relevant, this will be dealt with locally by the national authorities.
- This paper has been written by researchers from Chatham House and further developed through consultation with the co-authors. In addition, a Nordic-Baltic reference group (see acknowledgements) has provided their written comments in a peer-review process. The manuscript was also submitted to an open public consultation in July 2022 to September 2022. Whilst numerous changes were made in response to comments received through the open consultation and peer-review process, the final version of the paper is the sole responsibility of the authors.

This review has been developed as a collaboration between the NNR2023 project and Chatham House. A group of Nordic and Baltic scientists have given significant scientific input, whilst the members of the NNR Committee have ascertained the relevance is within the scope of the NNR project. This paper, in addition to several forthcoming papers and other major reports, will serve to assist the NNR Committee when formulating scientific advice to the authorities (Box 1).

## Food systems within the earth system

### The food system

The food system is a ‘complex system’. Complex systems typically exhibit non-linear behaviour, meaning they respond in different ways to the same input depending on their state or context. Non-linear systems are those where a change in an input driver does not necessarily produce the same proportional change in output. In the food system, such non-linear behaviour arises from economies of scale driving feedbacks: for example, intensifying grain production drives up the scale of grain production, driving down the price, making it economically possible to utilise grain for animal feed, making meat cheaper, stimulating demand for meat and ultimately allowing accelerating consumption of meat-based products (19, 20).

A commonly used definition of the food system was published by the Food and Agriculture Organization’s (FAO’s) High Level Panel of Experts on Food Security and Nutrition (HLPE) as:

All the elements (environment, people, inputs, processes, infrastructures, institutions, etc.) and activities that relate to the production, processing, distribution, preparation and consumption of food, and the output of these activities, including socio-economic and environmental outcomes. (21)

A similar definition of the food system has been published by FAO (22). We expand on this definition in Box 2.

Box 2 Defining a ‘food system’.

The term ‘food system’ encompasses the entirety of the production, transport, manufacturing, retailing, consumption and waste of food. It also includes impacts on nutrition, human health and well-being, and the environment, culture and social and economic aspects. Food security is a function of variations in the food system in any given location and is influenced by a range of socio-political factors affecting price, availability and access. Whilst there is an overall global food system (encompassing the totality of global production and consumption), there are also many subsystems within it. Each location’s individual food system is unique and is defined by that location’s mix of food produced locally, nationally, regionally or globally.

For each product consumed, there is a supply chain, which describes the way food and its ingredients get to consumers. The term value chain describes the mechanisms through which the value of a product is increased by transport, processing and packaging along the supply chain. The term ‘food system’ includes all supply chains (and, implicitly, value chains) as well as their impacts on the environment and people. Food systems inherently incorporate feedback, leading to direct and indirect effects; in turn, this can create feedback loops wherein the system responds in unexpected ways to small changes in the forces acting on it. Food systems are, therefore, dynamically changing systems; thinking only about supply chains and value chains is unhelpful both analytically and for policymaking, as it avoids consideration of wider system dynamics.

All activities within a food system – whether production, processing, retail or cooking – have impacts on the environment. For example, land under agriculture is disturbed from its natural state, which affects soils, water, biodiversity and even local microclimates. Processing, transport and retail require energy, water, infrastructure (e.g. roads) and other inputs – e.g. packaging. Throughout, pollution comes from chemical usage and disposal (e.g. from fertilisers, pesticides, antibiotics, industrial processes and greenhouse gas (GHG) emissions) as well as from the disposal of waste, including plastics and other packaging.

### Environmental systems underpin planetary health

Ultimately, food production depends on natural resources, e.g. land, soils and water – and often the embedded biodiversity that supports production, from microbes in the soil to pollinators and the ‘natural enemies’ that play a role in regulating populations of pests. Inevitably, food production impacts on the environment through three main routes.

**Fig. 1:**
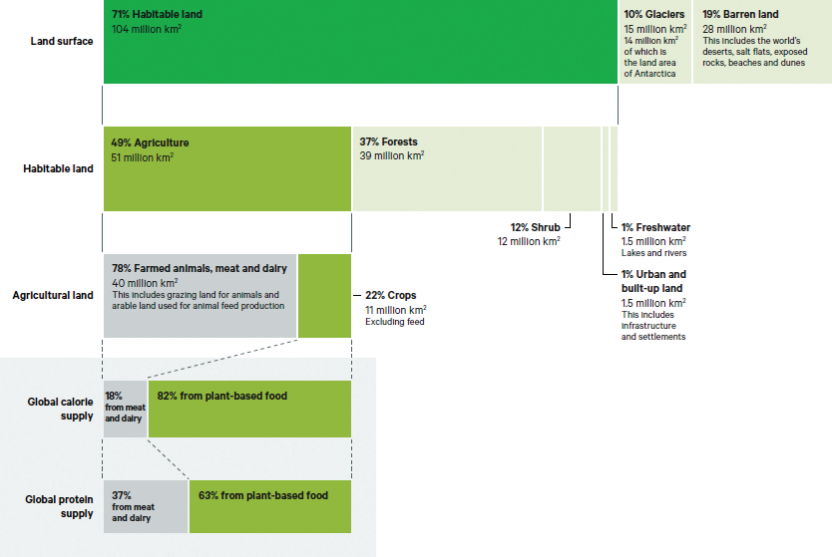
Global land ‘foodprint’. Notes: Agricultural land is the sum of arable land, permanent crops, permanent meadows and pastures. Permanent meadows and pastures is the ‘land used permanently (5 years or more) to grow herbaceous forage crops, either cultivated or growing wild (wild prairie or grazing land)’. Image source: Benton et al (13), modified from Ritchie and Roser (23). Data source: UN FAO. All visualisations, data and code produced by Our World in Data are completely open access under the Creative Commons BY license.

First, in terms of the direct impacts on land – both land under agriculture and through incentives that stimulate more land being brought into agriculture – so that agriculture is the predominant form of global land use (Fig. 1). As land is converted, blocks of natural land are reduced in size and are also fragmented. This may reduce the amount of space for nature and has a direct impact on ecosystems and the way they function (including carbon storage, water and climate regulation and biodiversity). However, fragmentation and loss of natural land cover can also have a significant indirect impact on ecosystems through ‘ecological rewiring’. As ecosystems tend to be a co-evolved set of interacting species that operate in equilibrium, a loss or reduction of even a single species can destabilise the whole ecosystem.

The second major route for impact – beyond habitat loss and fragmentation – is via the degradation of habitat, often via the intensification of agriculture (increasing yields per unit area). This impacts on environmental functions through reducing small patches of non-managed land (as fields become amalgamated into monocultures) and degrading the quality of farmland in general, which limits opportunities for biodiversity. Primarily, this is through reducing heterogeneity of habitats and focussing on ever larger-scale monocultural production – whether single crops or herd sizes. The associated inputs of nutrients and pesticides reduce the ability of most species to live there. Nutrient run off into ditches, streams and rivers, can lead to environmental changes occurring very far away, geographically, from agricultural land (13).

The third major route is via food systems driving climate change. Globally, the food system is responsible for more GHG emissions than any other aspects of our economies^II^. According to the Intergovernmental Panel on Climate Change (IPCC) (24) (see Table 1), food systems account for about one-third of all GHG emissions through GHGs from land use change and directly from agriculture (e.g. enteric fermentation, fertiliser, manure management and soil degradation). The financial incentives to agriculture both directly drive habitat loss and degradation and also drive climate change, indirectly driving habitat loss and degradation. Food systems therefore are intimately linked and interacting drivers of ecosystem disruption.

**Table 1:** Greenhouse gas emissions (GtCO_2_-eq yr^-1^) from the food system and their contribution (%) to total anthropogenic emissions. Mean of 2007–2016 period

Food system component	Emissions (Gt C0_2_eq yr-^1^)	Share in mean total emissions (%)
Agriculture	6.2 ± 1.4^a,b^	10-14
Land use	4.9 ± 2.5^a^	5-14
Beyond farm gate	2.6^c^-5.2^d^	5-10^e^
Food system (total)	10.8-19.1	21-37

Source: IPCC (24). Notes: Food system emissions are estimated from 1) FAOSTAT (2018), 2) US EPA (2012), 3) Poore and Nemecek (2018), 4) Fischedick et al. (2014) (using square root of sum of squares of standard deviations when adding uncertainty ranges; see also Chapter 2); and 5) rounded to nearest fifth percentile due to assessed uncertainty in estimates. Percentage shares were computed by using a total emissions value for the period 2007–2016 of nearly 52 GtCO2-eq yr–1 (Chapter 2), using global warming potential (GWP) values of the IPCC AR5 with no climate feedback (GWP-CH4 = 28; GWP-N2O = 265). This table is reproduced here in accordance with the IPCC copyright: Copyright – IPCC.

### Complex systems and complex dynamics: non-linear responses and thresholds

The reality of the non-linear behaviour of complex systems means that their behaviour changes unevenly even if input drivers are changing at a constant rate (i.e. are linear). Non-linear behaviour implies the existence of thresholds or boundaries, below which a system can cope, and above which it cannot. For example, as populations decline – through, for example, habitat degradation – the inevitable result is that individuals become rarer. At a certain level, this means it becomes difficult to find a mate, and therefore, reproduction rate decelerates, rarity further increases and the population accelerates towards extinction: populations often decline slowly and then ‘crash’.

Non-linear behaviour also applies to ecosystems, which are often ‘re-wired’ by environmental change caused by humans. Such ecosystem rewiring typically causes cascading impacts that often destabilise the whole ecosystem. For example, habitat fragmentation tends to lead to the loss of ‘keystone species’ such as large carnivores. For less mobile species, the habitat may remain plentiful, but cascading impacts arise as carnivory declines and herbivory increases, leading to changes in species abundances including outbreaks of pests. Another example is nutrient enrichment of water-courses adjacent to agricultural lands. Natural systems tend to be nutrient-limited, so when excess nutrients are added, the species most competitive for nutrients thrive – often algae or other microbes. Blooms of such species can shade the water and use up its oxygen – a process called eutrophication, which can lead to the collapse of biodiversity within the natural ecosystem. Excess nutrients can also be dispersed downstream into lakes and coastal zones and cause further algal blooms and hypoxic dead zones (25, 26).

Non-linearity is also important in climate change. The Paris agreement aims to keep anthropogenic warming well-below 2°C above pre-industrial levels to reduce the risk of passing ‘tipping’ points – thresholds beyond which positive feedbacks (such as the release of methane from melting permafrost causing more heating and in turn more permafrost melt and methane release) make it difficult to return to below the threshold. These include the potential for loss of ice sheets, which, once ice-sheet loss is triggered, will continue to melt over the next centuries and may cause considerable sea level rise (during the last period of warming, the mid-Pliocene Warm Period– when global CO_2_ in the atmosphere was similar to today’s and there was 2–4°C warming above the 20th Century climate – sea levels were up to 25 m higher than today (27)). In addition, increased thawing of the arctic may lead to the release of methane at rates that accelerate climate change, and for which, mitigation may not be possible.

### Complex systems’ implications for ‘sustainable food’

Recognising that the food system is a complex system operating within the complexities of the earth system, we identify five key points to consider. First, there will be thresholds in the way that food systems can absorb environmental degradation, and thresholds in the way that the impacts of food systems can be absorbed by the wider environment. Second, altering the food system (by changing some aspect of demand or supply) will have a range of consequences, some unintended. Third, there are many ways that the environment can be impacted by food system activities, and such impacts might be prioritised or traded off against other impacts under different circumstances. Fourth, the same activity can have different outcomes in different contexts; and fifth, the environmental impacts of food production and the place of consumption can be very far apart, leading to challenges of understanding, measuring and managing, the totality of the impacts across multiple countries. We highlight each of these, in turn, in the following sections.

#### Local and planetary boundaries will exist due to non-linear behaviour from complex systems

As more and more is produced and extracted from the same land, or more pollution is released into land, water or air, it is likely that the system will ‘gradually, then suddenly’ lose functionality (28). This implies that there will be upper-limits of production, below which the system works and above which the system breaks. Hence, there is no absolute recipe for ‘sustainable food’: what is sustainable at one place or time may be unsustainable at another place or time. Whilst defining local and planetary boundaries is challenging, partly due to large uncertainties (see below), the planetary boundaries concept is essential for understanding the environmental limits within which food systems should operate.

#### There will be unintended consequences of action

Any intervention can have impacts that propagate through the system via multiple routes; therefore, there will be unintended consequences of action. A prime example might be decreasing the GHG intensity of dairy production (reducing GHG emissions per unit of product), through increasing intensity and productivity. This can increase profit and reduce the cost of milk, stimulating further demand, incentivising the growth of the sector and leading to the increase in absolute emissions whilst reducing the relative emissions intensity of a product.

#### The multiple components of sustainability and their trade-offs

There are multiple ‘axes’ of environmental sustainability, including the impacts on land, soil, air, water, climate and biodiversity. This list can be expanded to include a range of other factors that play an important role in overall sustainability, i.e. expanding the remit of sustainability beyond environmental issues. Such factors include animal welfare, social factors, e.g. farmer and farm workers rights and welfare (29), and universal rights to access healthy and sustainable food (30, 31). In the report from 2019, the WHO and FAO propose that ‘Sustainable Healthy Diets are dietary patterns that promote all dimensions of individuals’ health and wellbeing; have low environmental pressure and impact; are accessible, affordable, safe and equitable; and are culturally acceptable. The aims of Sustainable Healthy Diets are to achieve optimal growth and development of all individuals and support functioning and physical, mental and social well-being at all life stages for present and future generations; contribute to preventing all forms of malnutrition (i.e. undernutrition, micronutrient deficiency, overweight and obesity); reduce the risk of diet-related noncommunicable diseases (NCDs); and support the preservation of biodiversity and planetary health. Sustainable healthy diets must combine all the dimensions of sustainability to avoid unintended consequences’ (32).

These factors trade-off against each other in many circumstances and in a variety of ways (33, 34). For example, increasing the ‘carbon efficiency’ of a meat product implies increasing the intensity of production and production methods, which may lead to adverse impacts on water and air quality, land and biodiversity through an increased demand for (and thus supply of) animal feed crops (29). Conversely, measures that aim to improve animal welfare by increasing space provision either through larger indoor space allowances, outdoor rearing and/or by extending the lifetime of animals all reduce productivity and thus result in higher ‘GHG footprints’ relative to animal-sourced foods from more intensive production methods (35). Similarly, farming methods that aim to minimise adverse impacts on biodiversity or provide more opportunities for biodiversity within a farmland area may as a consequence reduce some aspects of efficiency (e.g. land use), creating a relatively large footprint in some dimensions (36). Given the multitude of trade-offs, it is important to expand environmental impacts beyond a food’s direct GHGs and instead incorporate a broad range including resource use (water, land, fertilisers, pesticides and antibiotics) and environmental impacts (such as biodiversity/habitat loss, land degradation, antimicrobial resistance, particulates and chemical pollution) (37).

Including a broader range of factors, such as animal welfare considerations, within the remit of sustainability brings with it additional complexity in measuring such factors in a comprehensive and meaningful way. Determining the bounds of sustainability is less problematic from a purely resource use perspective as factors such as land use and nitrogen use can be assessed within the context of earth’s biogeophysical and biogeochemical limits, and the usage levels that are possible to continue into the long term, across future generations, without reaching or surpassing those limits. Animal welfare measures tend to relate to space provision and/or access to outdoor space. The same determination of sustainability is not possible with such factors as they are difficult to define objectively, although it is possible to develop methodologies to measure and incorporate them into FBDGs (38). The way that different aspects (e.g. biodiversity vs pollution vs welfare) are weighted to assess sustainability is a normative judgement rather than a science-based one.

#### Context dependency of environmental impacts

In addition to the issues related to trade-offs and measurement complexity, context-dependency is common in relation to food’s environmental impacts (39). The same intervention (e.g. organic farming) can have better or worse outcomes depending on the management of the farm, the place (e.g. biophysical environment) and the neighbours (a conventional farm surrounded by organic neighbours can have the same biodiversity on it as an organic farm surrounded by intensive neighbours) (40).

Part of the context dependency of impacts arises from the non-linear behaviour of environmental and food systems. Converting an acre of land to agriculture makes relatively little difference if it is part of a larger pristine landscape but would make a big difference if it is the last acre of habitat in a landscape. Likewise, an intensive patch of land has a less adverse ecological effect at a small scale than a landscape scale (which may preclude species moving between foraging and nesting sites, for example). From a food systems’ perspective, a single farm in a region may have market-access problems, but a multitude of the same farm types may stimulate market infrastructure (40). Similarly, the timeframe associated with the production also effects, which impacts are accounted for in a food’s footprint and how they are attributed. For example, beef produced on freshly deforested land for local consumption in the Amazon rainforest area would be associated with much higher GHG emissions due to land use change impacts compared to beef produced in the UK for local consumption on land that was deforested many years before – despite both products being produced using the same production methods (for example, outdoor grazing). These frequency-, scale- and time-dependent outcomes mean that the same action has different consequences depending on when and where it happens. Hence, solely relying on production-standards-based approaches, such as outdoor grazing, as a sustainability indicator in some cases can only ever act as a proxy for sustainability, in turn, making system level assumptions with regard to production methods challenging.

#### Spillovers and the role of trade

National food consumption never maps entirely to national food production (because a significant amount of what may be produced nationally may be exported, and a lot that may be consumed nationally may be imported). This leads for the potential for significant spill-overs across geographies. For example, if a country decides to conserve its own biodiversity through changing its agricultural production in an environmentally friendly way, the cost of production in that country will typically increase relative to elsewhere (since the inputs and practices on which the ‘cheaper food’ paradigm depends will likely be reduced or avoided). Higher costs may drive down demand for the food in question produced in that country, but, if total demand stays the same, price signals through international market linkages will incentivise either greater use of inputs or the conversion of additional land somewhere else to compensate, and demand will be filled through trade. This potentially leads to a biodiversity ‘saving’ in one place, arising through environmentally friendly farming, but a biodiversity ‘cost’ in another, through intensification or land-use conversion, to meet demand for cheaper food via imports (41). Carbon leakage can also occur, for example, if the GHG emissions from locally produced meat within domestic GHG inventories ignore the GHG costs of producing and transporting imported feed.

## Approaches to assessing the environmental sustainability of food, diets and food systems

The methods used to assess the environmental impacts of foods (and thus diets) differ substantially from methods used in health and nutrition. Research on the health outcomes of food intake includes a large number of 1) intervention studies, 2) cohort studies and 3) mechanistic studies, whose results are summarised in qualified systematic reviews from which general conclusions are drawn (42–44). In contrast, the environmental impacts of food are commonly entirely modelled, using first a number of different empirical models to calculate e.g. emissions of methane from cattle eating a certain type and amount of feed and emissions of nitrous oxide from soils, and then aggregating these different results to the final impact of the food, the diet or the food system. Measuring the actual (point source) impacts in each case is often impossible (e.g. GHG emissions from global food production) or very expensive (e.g. measuring gaseous emissions from a large number of fields). Uncertainties in models for assessing emissions from these biological processes can be substantial (45). However, due to the methods having formal (mathematical) parts, some aspects of environmental modelling are certain. For example, 1 kg of wheat will always have a considerably lower environmental impact than 1 kg of pork meat if it requires 4 kg of that same wheat to produce the 1 kg of pork.

For modelled environmental impacts, statistical inference methods like confidence intervals and hypothesis testing do not apply (46) – methods that are central in health and nutrition research. However, uncertainty analysis can be performed using e.g. Monte Carlo simulation, or sensitivity analysis in which results are checked for how sensitive they are to model and data choices. This is central to all environmental modelling. Results from independent studies can also be summarised in meta-analyses as for nutrition research, but for many foods and environmental impact categories, the number of studies is still limited (47, 48).

The two main ways to estimate environmental impacts of diets are via Life Cycle Assessment (LCA) by measuring the impacts of individual foods and multiplying by total consumption in the diet, or by using mass/nutrient flow models to calculate the impacts of the entire food system (49–51). It is then possible to assess using either approach how current diets relate to a given threshold or boundary, such as climate change targets, i.e. GHG emissions levels that accord with 1.5°C of warming. Such boundaries or thresholds can also be used to identify which ‘future’ or theoretical diets accord with them, using LCA or mass flow models, i.e. by constraining environmental impacts through changes in demand or food waste reduction. Additional constraints to the diet can be included such as meeting nutritional and health requirements. Simplistic proxies of sustainability can utilise partial outputs from either approach to equate diets with sustainability – although the addition of boundaries or thresholds is not applicable to such proxies. We summarise each of these approaches and their limitations in the following sections.

### LCA approach to assessing relative impacts

To assess the environmental impact of a certain food product, a well-established, standardised and commonly used method is LCA (52, 53). In a LCA, a ‘bottom-up’ approach is used to quantify the emissions of environmentally damaging substances (e.g. gases such as carbon dioxide or pollutants such as nitrate and toxic substances) and the use of resources (e.g. energy, land, materials and water) from all processes associated with the production, use and disposal of the product. Environmental impacts can be reported as different ‘impact categories’ or ‘mid-points’, e.g. climate impact, eutrophication, eco-toxicity, energy, land and water use, or aggregated into one or a number of ‘environmental scores’ or so-called ‘end-points’. For example, to calculate the overall climate impact of a food product, the different GHGs (most importantly carbon dioxide, methane and nitrous oxide) are weighted together most commonly using a metric called the Global Warming Potential, which considers both the warming effect of different molecules and their lifetime in the atmosphere (54). Similarly, eutrophication potential is calculated by considering how different eutrophying substances (e.g. ammonia and nitrate) contribute to biomass growth. For water use, it can be disaggregated into blue (fresh, ground- or surface-water) or green (direct precipitation or stored in soil) water to indicate the source and weighting factors. Considering water scarcity can also be used to better reflect impact on local water resources. A number of more or less sophisticated methods are available for aggregating emissions and resource use into impact categories, which all function as proxies for actual environmental damages. The choice of impact assessment method is value based, and there is an on-going scientific debate about the appropriate metrics to use for different purposes (55, 56). The most commonly assessed environmental impact categories for food products to date include the climate impact (also called carbon footprint), the land, water and energy footprint, and the eutrophication and acidification potential (49). It is less common but possible to report other impacts such as particulate matter and biodiversity.

LCAs of food products typically include the production of inputs (e.g. fertilisers, pesticides, fuels and electricity), processes at farm level (e.g. the use of energy, emissions from soils and animals) or from fisheries (e.g. fuel use in boats), processing, transport and packing. Functional units within an LCA define the unit being assessed, for example a kilogram of environmental impact (such as nitrogen) incurred per kg of food produced, and the system boundaries define where the impact is being measured, for example from the farm to the retailer. Depending on the purpose of the LCA, some measurements stop at the farm-gate and some include additional life cycle stages such as food preparation and waste management. For example, if the purpose of the LCA is to compare different feeding strategies for pigs, including post farm stages is not necessary.

**Fig. 2:**
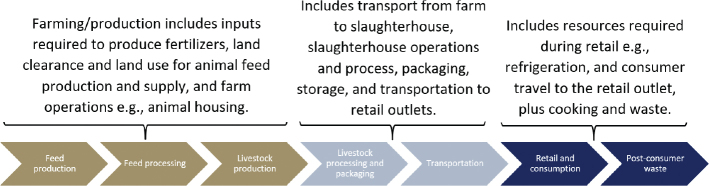
Example life cycle of a livestock product. Source/credit: Authors own.

Figure 2 shows an example of the lifecycle of animal-sourced foods, starting with the impacts of feed production and ending at the point of post-consumer waste. An LCA of food can also include environmental impacts (e.g. GHGs) associated with land use change (such as deforestation as a result of converting forest to agriculture for feed crop production), and additionally, for animal sourced foods, GHGs associated with manure and directly emitted from farmed animals.

There are several types of LCA. One important distinction is between attributional and consequential LCA. Attributional LCAs estimate how much environmental impact is a result of the product studied. A consequential LCA estimates the environmental burden of production and use of the product. For food commodities, attributional LCAs are most commonly used. An important difference is that attributional LCAs assess the impact from a static technosphere based on average data that represent the actual physical flows, whereas consequential LCAs assess the dynamic technosphere that reacts to a change in demand for different products. Another major difference between attributional and consequential LCA is how emissions are allocated between co-products. In attributional LCA, it is most often based on economic or physical relationships, whilst in consequential LCA, the system is expanded to include processes that are affected by the by-products entering the market. In attributional LCA, the environmental impacts related to the rearing of animals, slaughter and product processing are allocated to each product according to the economic value. If beef meat accounts for 70% of the overall economic value of the products derived from the animal, 70% of the life cycle impacts of that animal would be allocated to beef meat. In consequential LCA, the system is typically expanded to include alternative ways of producing the co-products, such as beef meat and leather. These could be, for example, producing beef in a system purely for meat production, i.e. from a suckler herd, and producing synthetic leather from biomass. The environmental impacts from these additional systems are then subtracted from the impact of the dairy system, leaving the impacts from the milk isolated.

It is also possible to differentiate between input–output LCA and processed-based LCA, which relates to how data from the different life cycle stages are collected. Input–output LCA is a top-down approach that uses economic input–output databases for data collection, e.g. the total economic activity and its associated emissions is divided between different sectors and then allocated further to products. Processed-based LCA is a bottom-up approach, describing the system being studied and mapping and assessing the emissions and resource use from the ‘ground up’.

LCA impacts are reported in relation to a ‘functional unit’ – which, for food commodities, is typically weight (e.g. kg CO_2_e/kg wheat), protein (e.g. kg CO_2_e/kg wheat protein) or energy (e.g. kg CO_2_e/1,000 kcals wheat). However, there are also studies that attempt to relate the environmental impact of foods to their nutritional content using nutrient density scores (57, 58).

**Fig. 3:**
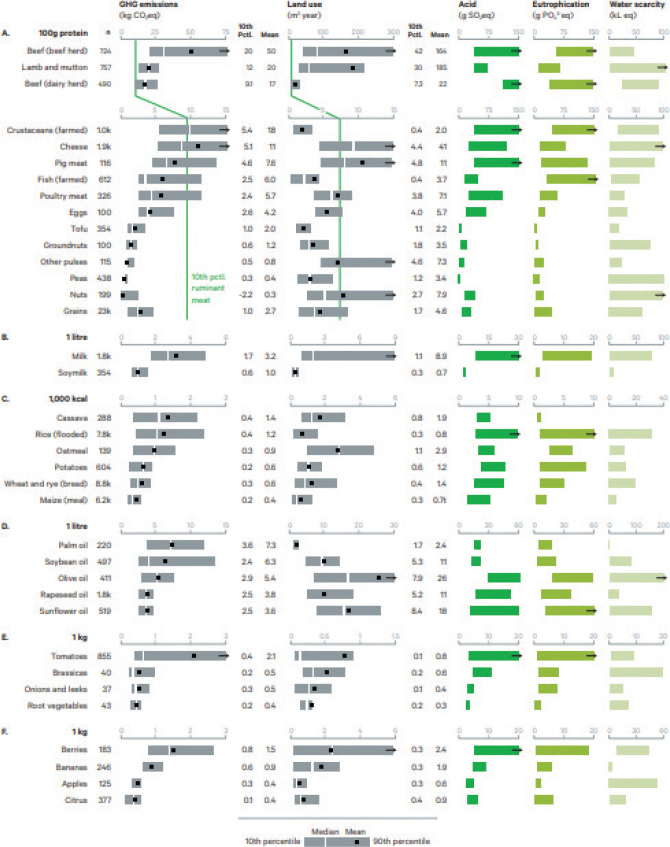
Relative environmental ‘footprints’ of a range of food products (13, 49). Notes: (A) Protein-rich products. Grains are also shown here, given that they contribute 41% of global protein intake, despite lower protein content. (B) Milks. (C) Starch-rich products. (D) Oils. (E) Vegetables. (F) Fruits. *n* = farm or regional inventories. Pctl. = percentile. Data source: Poore and Nemecek (49). Image source: Benton et al. (13).

Comparing and aggregating LCA data across different studies requires adjustments in order to harmonise methodological aspects such as functional unit and system boundary (59). Examples of environmental impacts from a range of food LCAs that were adjusted to the same system boundary and functional unit to enable comparison are shown in Fig. 3. The figure illustrates the substantial differences between food groups, e.g. tofu has on average around 25 times less GHGs per unit of protein in comparison to beef produced from a beef herd. There are also large variations in environmental footprints within the same type of food, e.g. within beef from beef herds, and between beef from beef herds and dairy herds.

**Fig. 4:**
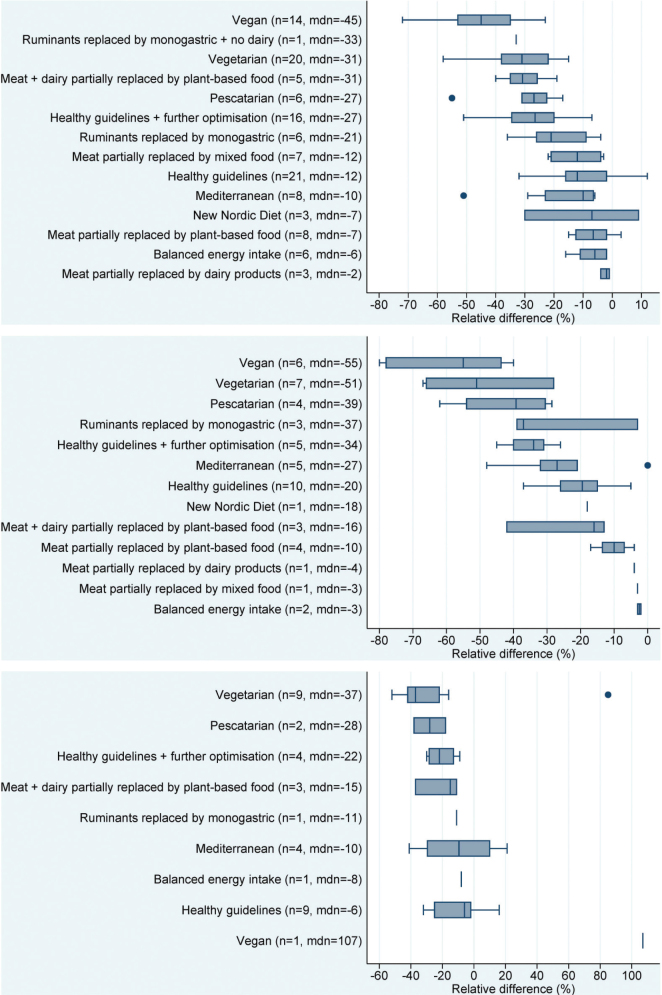
Changes in climate impact, land use and water use of various ‘sustainable’ dietary patterns compared to current average diets (48). Source/credit: Aleksandrowicz et al. (48). Figure included here is in its original format.

LCA data can be used to assess the environmental impact of diets by multiplying the LCA results for different foods by the amounts of foods in diets. Diets can be based on a national average from food supply data, from national dietary surveys (60), individual diets collected from dietary surveys or cohort studies (61, 62), or constructed diets e.g. diets that meet the given health/nutrition criteria (63, 64). The advantage of assessing whole diets compared with individual food products is the consideration of total volumes of food consumed. Reviews of such studies generally show considerably lower climate impacts and land use from diets with a lower content of animal-based foods, e.g. vegetarian or, in particular, vegan diets (47, 48, 65) (Fig. 4). The majority of studies included GHG emissions and land use (50, 66), although a number of studies also included other impact categories such as potential loss of biodiversity, and phosphorus and nitrogen use (60, 67).

### Aspects to consider when using the LCA approach

There are a range of aspects to consider relating to the use of food LCAs that are relevant regarding their usefulness in the context of dietary guidelines. There are a number of issues with the LCA methodology itself and the usefulness of its outputs in relation to determining the environmental sustainability of food commodities. First, there is the question of where to start and end the supply chain. Figure 2 shows the supply chain starting with ‘feed production’ and ending at ‘post-consumer waste’. However, it is not always clear which elements of the life cycle are included in individual studies, and the beginning and end points of the life cycle are not always consistent between studies, meaning cross-comparison is sometimes impossible without retro-adjusting the LCA start/end points accordingly.

Second, LCA focusses on relative impacts, i.e. it is a measure of ‘environmental efficiency’, and typically, it is difficult to draw absolute or systemic conclusions from it. Whilst a kg of beef has a much larger LCA footprint compared to a kg of chicken across a range of environmental metrics, if chicken was produced in large enough quantities, the aggregate impact of the chicken sector could be larger. This is exemplified by the ‘Jevons Paradox’.^III^ For example, the GHG impact of chicken production in Sweden was reduced by 20% between 1995 and 2006, but overall consumption – and therefore production – increased much more, completely off-setting the reduction. Recently, however, methods to relate LCA results to ‘absolute’ limits have been suggested (68), but these have not yet reached widespread use. Such methods are also associated with a range of value based and ethical choices that need more discussion. Related to this, relying solely on LCA for informing dietary guidelines would not provide a target or limit to design the guidelines around. For example, to reduce the impact of meat consumption, a shift from beef to chicken could appear to be favourable based on LCA data (depending on which indicators are included and how they are weighted). However, without having an overall target (such as a 50% reduction of food-related GHGs), it is not possible to assess whether such a shift would be adequate, or if further measures would be required. Similarly, without a whole diet GHG ‘budget’, for example, based on planetary boundaries, it would not be possible to assert whether a shift from beef to chicken would be adequate to stay within such boundaries when all other foods were also included. In addition, without a target or budget, the Jevons Paradox issue could emerge, where lower impact foods are substituted for higher impact foods and subsequently consumed in greater quantities – negating GHG savings.

Third, as the goal of LCA is to isolate the environmental aspects of individual products, it cannot capture system effects of the highly complex and interconnected food system. Many LCA-studies show favourable effects of replacing imported soy used for animal feed with locally grown protein feeds or e.g. insects produced on waste streams. However, the abolishment of soy for feed would have broader food system-level consequences. For example, replacing all imported soybean meal with local protein crops would increase the cropland devoted to animal feed production, reducing the area available for food production. Such a strategy might unintentionally shift import dependency from feed crops to food crops, increasing land demand elsewhere, with potential negative environmental effects.

Fourth, some aspects of environmental performance are more easily assessed, others are not (a limitation of environmental modelling in general). This tends to lead to a focus on the environmental aspects that are more easily modelled (e.g. GHG emissions or energy use) and a lack of focus on those that are not (such as eco-toxicity and impacts on biodiversity). Even if impacts are known and measurable and measured consistently – they could lead to a situation of comparing impacts on water use versus impacts on biodiversity loss, for example. Context specificity will be important to navigate such trade-offs – thus requiring more information beyond the scope of LCA.

Fifth, the variability in environmental impact between site- and region-specific production systems and the impacts of scale are also important to consider. To capture such differences, there is a need for site and region-specific data, which is resource demanding and difficult to access. In addition, the availability of food LCAs differs between food items and categories. For some foods, there are still data gaps, which may challenge environmental assessments of complete diets.

In summary, to date, most LCAs present only a portion of environmental impacts, and where those are presented, they are not always comprehensive. Hence, there are ‘gaps’ or inconsistencies across product impacts (such as some beef LCAs counting impacts associated with animal feed production, or land use change and others not), which makes comparison problematic, but also there are gaps in terms of the types of impacts measured. For example, most food LCAs measure GHGs but not necessarily land use change or biodiversity – this is largely because of the complexity in measuring such impacts and thus a lack of data availability or avoiding double counting (for example, if land use change impacts are allocated to a different sector). Partial assessment of impacts and the fact that LCA measures environmental efficiency without considering absolute boundaries could potentially lead to unintended consequences of actions taken. For example, shifting from beef to chicken production could reduce GHGs and land use overall but could increase requirements for intensively produced animal feed, increase the number of intensively farmed animals in the farming system, and subsequently increase antibiotic use and risk of new zoonotic diseases developing.

Despite the many challenges of LCA, the application of this method to assess the environmental impact of food has increased knowledge of the environmental sustainability of foods in several ways. Applying a life cycle perspective, i.e. including the impacts from all major processes associated with the production of a certain food, across all stages of the life cycle, has revealed hotspots within the lifecycle (the majority of impacts often arise in primary production, i.e. agriculture, rather than from processing, packaging and transport) and hotspots amongst foods (animal-based foods generally have considerably greater adverse impacts than plant-based foods) (49). What has also become clear is that uncertainty and variation can be substantial, highlighting why the magnitude, rather than detailed numbers, should be the focus when interpreting LCA-results.

### Approaches to assessing systemic impacts

#### Thresholds

**Fig. 5:**
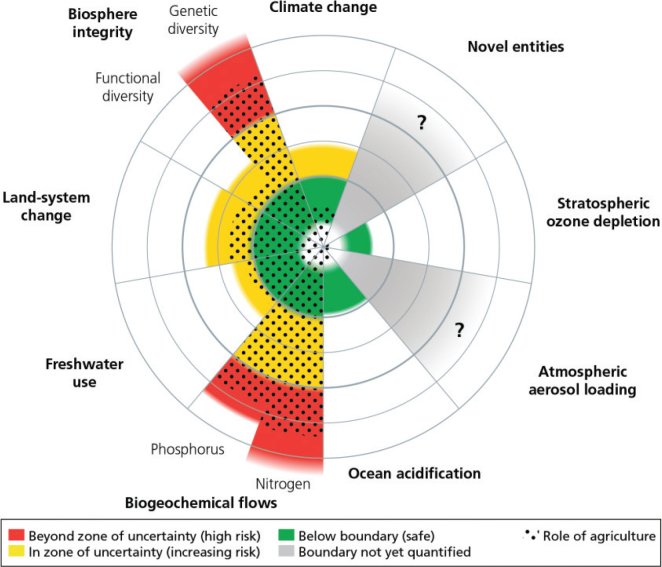
The current impact of agriculture in relation to planetary boundaries. Source/credit: Campbell et al. (70). Figure included here is in its original format.

Most studies to date assessing the environmental impacts from diets share the same limitation as with LCA of individual food products in that they do not commonly relate the results to any absolute limit, i.e. they compare diets with different food compositions but do not assess whether the diet is ‘good enough’ when scaled to the global level. However, some attempts have recently been made to do this (8, 14, 67, 69). The EAT-Lancet commission modelled the environmental impact of a healthy dietary pattern using LCA data, in combination with reduced food waste and production side improvements, and benchmarked against planetary boundaries (8). An earth systems approach was used to set the environmental boundaries for food production, i.e. boundaries that sustainable global food production must stay within to help create a safe operating space (indicated by the green area on Fig. 5). The EAT-Lancet boundaries present limits for the food system related to climate impact, land and water use, biodiversity loss and N and P use derived from the planetary boundaries framework (Fig. 5). Campbell et al. (70) estimated the role of agriculture (see black dots in Fig. 5) in the status of planetary boundaries on land-system change (80%), freshwater use (84%), nitrogen use (85%), phosphorous use (90%), biodiversity loss (90%), climate change (25%) and ocean acidification (25%). Whilst such analysis depends heavily on the global boundaries and the environmental impact used, the EAT-Lancet example demonstrates that it is possible to produce – at the global level – a diet that meets health criteria within the environmental boundaries, when diet shift is combined with production side improvements and reduced food waste. However, the uncertainty ranges in the EAT Lancet boundaries are considerable and notably high for extinction rate. Given the existence of databases, locally and globally, that collate LCA data, there is the potential to construct culturally appropriate local FBDGs taking a similar approach to the EAT-Lancet (e.g. x portions of food-type y per week).

#### Aspects to consider when using thresholds to assess systemic impacts

The ‘planetary boundary’ or limit on impact being applied is very important in determining dietary composition. For example, a less stringent boundary or limit on food GHGs could provide more allowance for GHGs per capita, increasing the inclusion of higher impact foods. Conceptualising planetary boundaries to levels that do not necessarily keep pace with or match the implications of today’s science in terms of the levels of GHG reductions needed to align with 1.5°C aspirations could be problematic in that dietary guidelines based on them would quickly become inadequate and in need of revision. Related to this point, conceptualising environmental or health boundaries from a political acceptability perspective or least cost pathway does not necessarily equate to earth’s biogeophysical or biogeochemical boundaries.

Using location-specific LCAs in a global analysis could be problematic in terms of allocating equal impacts to the same product produced in different parts of the world, which could result in under or overcounting impacts as they could be different in reality (71). Taking a local systems approach is also problematic as it does account for impacts at the global level, in terms of where those production impacts would occur and whether consumer impacts at the local level are sustainable within global planetary boundaries.

The challenges of LCA also impact the utility of thresholds that rely on them. For example, if impacts are not measured or are difficult to measure and associated with inaccuracies, this could result in a partial estimation of total impacts. In particular, data on land use footprints and energy use are relatively complete, and data on the impact of farming on biodiversity are very incomplete. Basing footprints only on what is easier to measure would, in such a case, bias considerations towards high intensity farming, rather than more extensive farming.

In addition, no standardised method exists to operationalise the planetary boundary framework to a given sector or system level, such as the food system. Therefore, different methods are used in different studies to define environmental boundaries as well as how emissions and resources should be allocated over time, between different activities and across the global population. Furthermore, underlying assumptions are needed, which will greatly affect per capita environmental boundaries (e.g. whether accounting for future global population growth, whether adults and children are allocated the same share of emissions/resources, or whether there is an equal or unequal per capita share across countries and hemispheres, irrespective of development status).

## Food system models

An alternative approach to combining LCA data with thresholds is food system models that calculate the environmental impacts from total production driven by a given demand for food. By doing so, they inherently cater for system effects (including joint production) and availability of land. Some models are purely biophysical models, and diets are in that case exogenously set, whilst some models include an economic demand system, which determines total consumption based on consumer preferences and food prices (according to elasticities of demand). Models can be global or regional, with the additional complexity of the latter needing to consider trade.

Schader et al. modelled a range of different global scenarios, of which we highlight two here (51). First, a ‘business as usual’ scenario without dietary change, focussing on the efficiency of agriculture in the sense of minimising emissions in products, led to a significant increase in arable land (as livestock switch to predominately grain fed) and the abandonment of grasslands for agriculture. This, in turn, had significant adverse impacts on nitrogen cycling, water-use and deforestation (incentivised for grain production). Second, a dietary shift to increase the consumption of plant-based foods, combined with a 71% reduction in protein intake from livestock products, resulted in a smaller water, nutrient and biodiversity impact – despite the environmental efficiency of livestock production being reduced compared with the business as usual scenario due to a shift to grassland-based feed for ruminants and use of by-products (waste) to feed non-ruminants. This study illustrates that systemic approaches can produce very different outcomes. LCA approaches: if people reduce meat consumption enough, grass-fed ruminant production can lead to better environmental outcomes than switching out of ruminants to grain-fed poultry. This result arises from a number of issues, including: (1) significant areas of land used for agriculture are more suitable for grazing/pasture than other agricultural uses, (2) extensive grazing can have positive impacts for some environmental metrics, like biodiversity and soil quality, (3) mixed farming (or landscapes) can enhance nutrient cycling through organic fertiliser usage, reducing synthetic inputs and (4) mixed farming landscapes are more heterogenous that can benefit biodiversity in a number of ways.

Hence, whilst an environmental efficiency measure – developed through LCA – is indicative of *relative* impacts, reduction in consumption of ruminant meat can lead to *absolute* reductions in emissions to the point that its relative efficiency is less important than the positive relationship between ruminant farming and some environmental metrics.

In summary, systems approaches attempt to model the entirety of the food system, including the dynamic relationships between demand and supply, and their aggregate environmental impact. As they are broad systemic models, they rarely have the granular detail of an LCA model of a single supply chain and a single product. As a ‘top down’ model, they are most useful for examining the systemic and dynamic aspects of the food system in aggregate. Assumptions about demand are often crucial determinants of system dynamics, environmental impacts, and therefore what food can be sustainably produced, and how it can be partitioned to ‘sustainable diets’. Assessing environmental impacts of diets within the context of thresholds is relevant to LCA and systems approaches to determine, for example, how a food system measured using a mass flow model relates to planetary boundaries.

### Aspects to consider when using food system model approaches

Whilst systems approaches are useful in numerous ways, there are some challenges to consider. Principal amongst these is a relative lack of use of such approaches in the academic literature (compared to LCA) and therefore a lack of methodological robustness in how to understand the aggregate impact of the food system on the environment, and how to minimise this whilst providing nutritious food for all.

There are also dynamic elements within a system that are difficult to capture. For example, projecting future dynamics requires making assumptions on the demand side, often in relationship to population growth and timeframe in terms of numbers, demographics, income and location – which would all impact food demand and production. Whilst this criticism also applies to LCA-based approaches because they do not aim to assess the absolute impacts, changing patterns of demand and supply are less problematic conceptually.

### Proxies for sustainable food

Simple proxies are often used, particularly in civil society discourse, in an attempt to define food as environmentally sustainable. Examples of such proxies relate to food miles (e.g. defining locally produced food as more sustainable than food imported from further away), food provenance or national self-sufficiency (e.g. all food consumed, or large portions of it, within a country is also produced within the same country), the farming system (e.g. organic agriculture or regenerative agriculture) or even more loosely defined proxies such as ‘real food’ or ‘naturalness’. Whilst proxies are simple in terms of focussing on one aspect such as transport or production method, this is their main limitation. There is no simple proxy that maps onto a sustainable food system. A focus on food miles or local production fails to account for a multitude of impacts, including major portions of a product’s LCA impact (e.g. ~90% of impacts occur during the production stage, with transport generally accounting for less than 10% of total impacts (49)). Using organic production as a basis for making food more sustainable could have unintended consequences, as more land and resources would be required to meet existing demand (at the system level, organic production is only feasible in conjunction with major demand shifts to more plant-based diets) (72, 73). Proxies also potentially distract from other changes that could have much bigger impacts. For example, shifting less than 1 day of energy intake per week from red meat and dairy products to chicken, fish, eggs, or a vegetable-based diet achieves more GHG reduction than buying only locally sourced food (74).

## Integrating health and environment

In this section, we consider the synergies across health and environmental impacts of food and diets.

### Commonalities across healthy and environmentally sustainable diets

There are some top-level common conclusions regarding health and environmental sustainability within the context of diet that can be considered well-supported by the scientific literature. Trophic relationships play a central role in terms of environmental impacts of diets. In essence, growing plants for direct human consumption has a lower environmental impact – certainly for GHGs and land use, and typically for eutrophication and acidification potentials – than growing plants on cropland for farmed animal feed to provide animal products for human consumption (see Fig. 3). A number of analyses (47, 48, 75–78) have demonstrated various savings associated with dietary shifts that benefit from simplifying trophic relationships in the food system (making a range of simplifying assumptions, particularly about linearity of the observed associations at a large scale). For example, Shepon et al. (79) demonstrated that rather than produce crops for farmed animal feed in the US (which accounts for 67% of total dietary energy production), if the cropland was reconfigured in a way that optimised food production for human health whilst using a minimum of environmental resources, more than twice the number of people (350 million) could be fed from the same area of land. More than a third of all dietary energy from crops produced globally are fed to farmed animals – only 12% of those return to the food system for human consumption (80). If crop dietary energy were instead provided for direct human consumption, an estimated 4 billion additional people could be fed (81).

The implications of such trophic relationships also impact other factors. For example, largely due to the greater requirement for land and nutrient pollution from producing animal sourced foods, they are generally associated with greater biodiversity losses compared to growing plants for direct human consumption (15, 82). Due to an expected increase in the consumption of animal sourced foods globally, they are also identified as not only major drivers of current environmental impacts such as climate change and biodiversity loss but also dominant future threats (15). The extent to which diets shift to less resource intensive foods also largely determines options for tackling major environmental issues, including climate change and biodiversity loss (13, 15, 17, 80, 83). Thus to a first approximation, at the system level, obtaining food directly from plant sources in preference to animal sources generally has a lower environmental impact.

A second criterion to consider is the diversity of products. In a high-income country context, if a diet rich in plant-based foods reduces the impact relative to one rich in animal sourced foods, then eating a diversity of plants – fruit, vegetables, pulses, nuts, leafy green vegetables and whole grain products – is likely to both be beneficial from a human nutrition (e.g. micronutrient and fibre) perspective and from a farming system perspective. It is well understood that many of the adverse environmental impacts – particularly on biodiversity but also on soils – arise from monoculture and simplified rotations. Diversified diets could enable more diversified agriculture, and more benefits arising for wildlife (13).

In addition to *what* is eaten, a number of studies highlight that significant ‘sustainability improvement’ arises from eating amounts consistent with energy requirements (i.e. to eat sufficient rather than excessive amounts) (8, 14, 17). Excessive energy intake is a form of food waste that has both an environmental footprint and adverse health impacts (84).

### Coupling environmental and health analyses

Numerous analyses of dietary shifts that reallocate food production for direct human consumption have demonstrated a range of environmental and public health benefits. For example, plant-based diets adopted at the global level have been estimated to deliver environmental benefits including a 70% reduction in food-related GHGs, a 76% reduction in the requirement for agricultural land, the removal of 547 Gt CO_2_ from the atmosphere through restoring native ecosystems on spared agricultural land (equivalent to 16 years of current global CO_2_ emissions) and a 49% reduction in eutrophication (49, 83, 85, 86). The public health benefits include reduced incidences of non-communicable diseases, including heart disease, cancer, stroke and diabetes, amounting to an estimated 10% reduction in global premature mortality rates and a resultant $30 trillion/year saving in healthcare costs (86, 87).

**Fig. 6:**
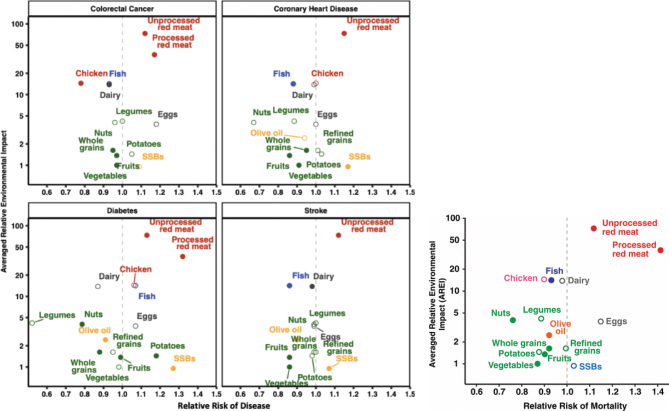
Association between a food group’s environmental impact and impact on human disease and mortality. Source/credit: Clark et al. (88). For the x axis on relative risk of mortality, a relative risk >1 indicates that consuming an additional daily serving of a food group is associated with increased mortality risk, and a relative risk <1 indicates that this consumption is associated with lowered mortality risk. The y axis is plotted on a log scale and is the averaged relative environmental impact (AREI) of producing a serving of each food group across five environmental outcomes relative to the impact of producing a serving of vegetables (not including starchy roots and tubers). For the x axes on relative risk of disease, a relative risk > 1 indicates that consuming an additional daily serving of a food group is associated with increased disease risk, and a relative risk < 1 indicates that this consumption is associated with lowered disease risk. Each graph shows a different disease. The y axes are on a log scale and are the AREI based on five environmental outcomes. Labels and points are coloured with green = minimally processed plant-based foods; dark blue = fish; black = dairy and eggs; pink = chicken; red = unprocessed red meat (beef, lamb, goat and pork) and processed red meat; dark yellow = sugar-sweetened beverages; and light yellow = olive oil. Food groups associated with a significant change in disease risk (at *P* < 0.05) are denoted by solid circles.

Coupled analyses for ‘nutritional impacts’ and ‘environmental impacts’ show some degree of correlation (see Fig. 6): i.e. a diet that has a lower environmental footprint can also be, to a first order approximation, one that is nutritionally adequate and health promoting (8, 14, 86). This suggests a ‘substitutability’ hierarchy (e.g. switch from red meat to fish/white meat, or better to plant-based foods) – shifting dietary choices from the top right quadrant to bottom left in Fig. 6 for maximum health and sustainability benefits. However, whilst this is potentially useful, there are likely to be trade-offs to consider in practice, given the many variances in food production impacts – even for the same type of food across different locations and production methods (as discussed in the LCA limitations section). For example, shifting from beef to chicken would reduce GHGs and overall land use but could increase food-feed competition and reliance on imported feed, bring more farmed animals into the food system, increase animal welfare concerns, increase risk of zoonosis and increase point source pollution. Conversely, a shift from beef or chicken to a plant-based food may not carry the same risks. Also, a shift within the plant-based food category is important to consider. Hence, whilst substitutability hierarchies have utility in some contexts, they do not take account of such system level impacts and trade-offs.

In a recent study, Clark et al. (88) paired ingredient lists for food products in the United Kingdom with environmental databases to derive estimated environmental impacts of 57,000 food products across four indicators: GHG emissions, land use, water stress and eutrophication potential. When comparing with the food’s nutrition quality, as assessed by NutriScore, they observed that more nutritious products are often more environmentally sustainable, but there are exceptions to this trend.

It is important to emphasise that coupled analyses for ‘nutritional impacts’ and ‘environmental impacts’ depend strongly on the health impact attributed to foods and food groups. Whilst the two examples discussed earlier are good examples of such analysis, much more extensive assessments of the health effects are needed based on qualified systematic reviews (see (42–44)).

### Methods for optimising diets for health and environmental sustainability

Analyses seeking to identify dietary patterns that minimise environmental impacts whilst meeting nutritional requirements and improving health have used scenario analyses and mathematical optimisation approaches. Computerised dietary programming (CDP) is one way to optimise diets with regard to several indicators and has been used to create new, more sustainable dietary recommendations and guidelines (89). In CDP, mathematical optimisation of diets is performed using linear or quadratic programming. Software programs handle data on several different variables, such as macro- and micronutrients, GHG emissions and land use and optimise diets based on minimising environmental impacts, e.g. GHG emissions, whilst meeting nutrient recommendations or keeping diets as similar as possible to a reference diet (commonly the current diet). The ‘Livewell plate’ produced by WWF was derived from linear programming to adjust the UK’s FBDG (the ‘Eatwell plate’) to one that minimises GHGs whilst meeting nutritional requirements (90). Similar approaches have been taken in academic studies. For example, Perignon et al. and Donati et al. (91, 92) used an optimisation approach similar to the Livewell approach, but across multiple environmental objectives, to suggest how diets could be adapted to maintain nutritional requirements but reduce environmental impacts across multiple dimensions. Such studies are often based on maintaining today’s dietary patterns, with a view to nudging people in more optimal directions and understanding the demographic correlates of who might make such shifts (93). In the EU project SUSDIET (ended in 2017), CDP (a tool developed by MS Nutrition in France) was used to develop nutritionally adequate diets with low GHG emissions that deviate as little as possible from current dietary patterns within the EU. Results from this project were published by Vieux et al. (94), where nutritionally adequate diets for each gender were developed using optimisation techniques starting from average observed diets (gender-specific) in five European countries (France, UK, Italy, Finland and Sweden) and applying stepwise 10% reductions in GHG emissions (66, 94). There are also studies that attempt to relate the environmental impact of foods to their nutritional content using nutrient density scores (e.g. (57, 95, 96)).

Apart from optimisation approaches, several other methods are used for combined environmental and nutrition/health assessments of diets (for examples, see (50, 91, 97–100).

## Key considerations for assessing the environmental sustainability of diets

In previous sections, we have demonstrated the complexity of assessing the environmental sustainability of foods and diets, and the limitations of the various approaches. Despite this, a pragmatic approach is needed in order to identify options for most effectively reducing environmental impacts whilst minimising unintended adverse outcomes. To assist this endeavour, we summarise the findings of the paper to identify five key considerations to guide assessments of sustainable diets (Box 3). They are not listed in priority order but are instead intended to provide a comprehensive list of considerations when integrating the environmental sustainability of food production and consumption into FBDGs.

Box 3 Five key considerations for assessing the environmental sustainability of diets.

1: **Consider the thresholds**. The limits of absorbing environmental impacts might vary depending on location and/or time but are always important to consider – as is the potential for shifting environmental burdens up or down stream (or leakage) to other areas. This implies there will be upper-limits of production, below which the system works and above which the system breaks. Boundaries (not just relative outcomes) need to be factored in, including some safety/error margin.

2: **Consider the system**. Apply a system perspective and use models that can capture such dynamics, such as the consequences of shifting an element of supply or demand on the entire system. This is important for avoiding an exceedance of environmental thresholds at the global level, for example if supply side efficiency gains were applied in conjunction with an increase in production amounts, this could result in an absolute increase in impacts at the system level, despite having lower relative impacts (i.e. per unit of production impacts would be lower).

3: **Consider the variables**. Assessments should include a wide range of aspects, extending beyond environmental impacts such as land, water and greenhouse gas (GHGs), to also capture important socioeconomic factors and how such impacts might be prioritised or traded off against each other under different circumstances. This is also important when assessing public health aspects, and the nutritional component of food to ensure, for example, that diets also meet nutrition criteria.

4: **Consider the context**. In addition to considering global perspectives, use evidence and perform research relevant to the local situation/context, and include appropriate stakeholders. As the same activity can have different outcomes in different contexts, it is important to refine the context accordingly to gain more accurate insights in terms of impacts.

5: **Consider the spillover**. Include impacts from production and consumption, i.e. not only territorial production-based impacts. The location of food production and its place of consumption can be very far apart in terms of distance (and sometimes across multiple countries), leading to challenges of understanding, measuring and managing the totality of impacts. However, acknowledging such ‘distant’ impacts of consumption is important to aiding understanding of the system level impacts.

## Footnotes

I The cheaper food paradigm drives supply and demand through a set of overlapping and often self-reinforcing mechanisms, with the primary goal of constantly increasing food production at increasingly lower costs, which involves converting more land to agriculture, and further intensifying farming methods. Consumption rises as food prices drop – resulting in more environmental degradation and disruption of our natural ecosystems, and climate change – leading to an increased requirement to bring fresh agricultural land into production. From: Benton et al. (13).

II This includes both direct emissions from agricultural production, indirect emissions from land-use change and emissions from transport and energy used along the food supply chain.

III Jevon’s paradox describes a situation where efficiency gains are offset or even lost due to a subsequent increase in consumption, driven by the benefits of the efficiency (such as a lower food price delivered through intensifying food production, in turn stimulating an increase in food consumption).
